# What Evidence Exists on the Effectiveness of Psychotherapy for Trauma-Related Distress? A Scoping Review

**DOI:** 10.3390/healthcare13233180

**Published:** 2025-12-04

**Authors:** Emma Victoria Shiel, Zoe Connor, Megan Downes, Abigail Bailey-Shaw, Steve Hemingway, Clare Walters, Susanna Kola-Palmer

**Affiliations:** 1 Mental Health Research Group, Division of Nursing, Social Work, and Midwifery, University of Manchester, Oxford Rd, Manchester M13 9PL, UK; 2 School of Health and Human Sciences, University of Huddersfield, Queensgate Campus, Huddersfield HD1 3DH, UKs.kola-palmer@hud.ac.uk (S.K.-P.); 3 The Walters Method, 46 Cockley Hill Ln, Kirkheaton, Huddersfield HD5 0HH, UK

**Keywords:** psychotherapy, trauma, intervention, distress, PTSD

## Abstract

**Highlights:**

**What are the main findings?**

- Eye Movement Desensitisation and Reprocessing (EMDR) and Cognitive Behavioural Therapy (CBT) are the most studied and effective treatments for trauma-related distress, with Prolonged Exposure and Narrative Exposure Therapy showing promise but requiring more research.
- Alternative psychotherapies, including psychodynamic approaches, demonstrate effectiveness in complex trauma cases (e.g., intimate partner violence, child loss) where EMDR and CBT may be less effective, though these remain understudied.

**What are the implications of the main findings?**

- Clinicians should consider a broader range of therapeutic options beyond CBT and EMDR, selecting interventions based on clients’ individual needs, cultural context, and trauma complexity rather than defaulting to the most studied approaches.
- The identified evidence gaps highlight the need for further research into alternative therapies and emerging approaches, like the Walters Method, which may offer accessible options for clients who find traditional evidence-based treatments challenging.

**Abstract:**

**Background/Objectives**: Trauma-related distress poses significant mental health challenges, with psychotherapy serving as a primary intervention. The Walters Method is a promising new alternative that may help where traditional methods fall short (i.e., in complex or violent cases), but before it can be implemented widely, the existing evidence on the effectiveness of other psychotherapies for trauma-related distress must be mapped to see how and where it relates to other techniques. The aim of this scoping review was to provide an overview of existing evidence on the effectiveness of psychotherapy for trauma-related distress. **Methods**: A scoping review was conducted to better understand the effectiveness of psychotherapies for trauma-related distress (including PTSD, acute stress disorder, or other serious mental health issues). **Results**: Thirty-three articles were analysed. Included articles included adults with PTSD, incarcerated women, childbirth trauma survivors, female survivors of sexual abuse, hospitalised COVID-19 patients, adults with serious mental illness, veterans and active soldiers, firefighters, and refugees. Eye Movement Desensitisation and Reprocessing and Cognitive Behavioural Therapy were the most studied and effective treatments. Prolonged Exposure and Narrative Exposure Therapy were less common but noteworthy. Other therapies, including psychodynamic approaches, are seldom studied but have proven effective when explored, highlighting knowledge gaps and potential missed opportunities. Success with these alternative approaches—especially in complex trauma cases like intimate partner violence or child loss where EMDR and CBT may be less effective—suggests they have potential, but further research is needed for validation. **Conclusions**: This review offers novel contributions to the field by emphasising innovative therapeutic perspectives that extend beyond traditional, more studied, evidence-based approaches such as CBT and EMDR, thereby expanding treatment options for diverse clinical presentations. Alternative therapies show promise, particularly for complex trauma cases like intimate partner violence or child loss where established approaches may be less effective; however, further research is needed to validate their efficacy across diverse populations. Selection of psychotherapy should be based on clients’ goals and comfort, and the cultural and contextual compatibility between the person and intervention. Future research should prioritise underexplored therapies to address current knowledge gaps and improve treatment accessibility for varied clinical needs.

## 1. Introduction

Trauma-related distress (defined in this study as a person’s emotional, psychological, and physiological response to a distressing and overwhelming event that disrupts their sense of safety and security) affects individuals worldwide and represents a significant mental health burden. Currently, there is a requirement for an accessible self-help toolkit that quickly reduces long-term emotional distress and motivates people to make positive life changes. Despite decades of research and clinical practice, contemporary trauma therapy faces significant challenges. Conventional approaches such as Cognitive Behavioural Therapy (CBT) and Eye Movement Desensitisation and Reprocessing (EMDR) have demonstrated effectiveness for post-traumatic stress disorder (PTSD), but show notable limitations when addressing complex presentations, including chronic trauma, developmental trauma, and cases involving multiple traumatic events. High dropout rates, limited accessibility, and the requirement for verbal processing of traumatic memories create barriers for many individuals seeking support.

In response to these limitations, there has been growing interest in body- and sensation-oriented therapeutic approaches. These include Somatic Experiencing, which focuses on releasing traumatic activation held in the nervous system; Sensorimotor Psychotherapy, which integrates somatic interventions with cognitive and emotional processing; and Gendlin’s Focusing [1], which teaches clients to attend to bodily felt senses of situations. The Walters Method (TWM, [2]), which belongs to this emerging field, is a ‘felt-sense’ [3] psychotherapy which aims to provide emotional freedom, could be the answer. TWM offers a unique approach by focusing on felt sensations and using guided meditations to uncover and address the root causes of emotional pain. This method does not require clients to talk about their issues, making it accessible and less intimidating for many individuals. By regularly practicing TWM, individuals can achieve greater mental clarity, emotional freedom, and spiritual growth. This toolkit could empower people to manage their emotional distress effectively and make positive changes in their lives.

However, before TWM can be implemented widely, a scoping review must be conducted to explore the existing evidence on the effectiveness of other psychotherapies for trauma-related distress. A scoping review methodology is appropriate for this purpose because the field is characterised by high methodological variability, diverse intervention types, inconsistent outcome measures, and heterogeneous trauma populations. Unlike a systematic review, which assesses study quality and synthesises effect sizes, a scoping review allows for systematic charting of the literature to map what is known, identify conceptual boundaries, and reveal gaps in the evidence base without restricting inclusion based on study design or quality criteria. This exploratory approach is necessary given the breadth of psychotherapeutic approaches and the complexity of trauma-related distress.

While there is limited evidence in this area, systematic enquiry is necessary to understand the specifics. Existing reviews focus on topics such as post-traumatic stress disorder (PTSD), but many are now potentially outdated (e.g., [4]) or focus on specific topics like childbirth [5].

For clinicians and mental health professionals, having a clear understanding of the effectiveness of different psychotherapeutic approaches could aid in making informed decisions about treatment options for their patients. This review aims to provide an updated understanding and a broad exploration of the effectiveness of psychotherapy for trauma-related distress as a whole. Conducting this review will map the existing data on this topic, helping researchers and clinicians understand where TWM stands in relation to other therapies. It will also determine whether TWM offers unique benefits and identify any care gaps. By synthesising the available evidence on psychotherapy, valuable insights can be provided for future practitioners and policymakers, informing clinical practice and guiding the development of evidence-based interventions like TWM for individuals experiencing trauma-related distress.

Finally, this review fosters collaboration between researchers, clinicians, and other stakeholders, which is considered good practice for developing comprehensive and effective approaches to addressing trauma-related distress. Additionally, this review may provide evidence to support a larger and more rigorous trial on TWM in the future.

This was an exploratory scoping review to identify what evidence exists on the effectiveness of psychotherapy for trauma-related distress. The goal was to chart the literature across a field characterised by high methodological variability and inconsistent outcomes, rather than to assess study quality. As this is an exploratory review, no expectations or hypotheses are reported.

## 2. Aims and Review Questions

The aim of this review was to provide an overview of existing evidence on the effectiveness of psychotherapy for trauma-related distress.

Two questions lead this research: (1) What evidence exists on the effectiveness for psychotherapy for trauma-related distress? (2) What barriers and facilitators have been identified in the literature for psychotherapy for trauma-related distress?

## 3. Materials and Methods

Since we are interested in charting the available evidence on the effectiveness of psychotherapy for trauma-related distress, it was felt that a scoping review was appropriate. A protocol was compiled using guidance for scoping reviews and registered in the Open Science Framework (https://doi.org/10.17605/OSF.IO/WGV9T).

A preliminary search of MEDLINE, the Cochrane Database of Systematic Reviews, and JBI Evidence Synthesis was conducted and no current or underway systematic reviews or scoping reviews on the topic were identified.

This review is reported using the Preferred Reporting Items for Systematic Reviews and Meta-Analysis (PRISMA-ScR) extension to scoping reviews (checklist available in Supplementary Materials). The following steps were adhered to:

1. Defining and aligning the objective/s and question/s.
2. Developing and aligning the inclusion criteria with the objective/s and question/s.
3. Describing the planned approach to evidence searching, selection, data extraction, and presentation of the evidence.
4. Searching for the evidence.
5. Selecting the evidence.
6. Extracting the evidence.
7. Quality appraisal (now recommended in JBI scoping reviews).
8. Analysis of the evidence.
9. Presentation of the results.
10. Summarising the evidence in relation to the purpose of the review, making conclusions and noting any implications of the findings.

### 3.1. Inclusion Criteria

The inclusion criteria for existing studies were developed using the ‘PCC’ (Population, Concept, Context) framework—a highly recommended search strategy framework for scoping reviews. The details are as follows:

#### 3.1.1. Population

Adults experiencing trauma-related distress. This criterion is broad, including but not subjected to conditions like PTSD, acute stress disorder, and other trauma-related serious mental health issues (SMIs).

#### 3.1.2. Concept

Effectiveness of Psychotherapy—any form of psychotherapy aimed at reducing trauma-related distress. This includes, but is not limited to, Cognitive Behavioural Therapy (CBT), Dialectical Behaviour Therapy (DBT), Eye Movement Desensitisation and Reprocessing (EMDR), psychodynamic therapy, and other relevant psychotherapeutic approaches.

#### 3.1.3. Context

Any setting where psychotherapy may be administered, e.g., clinical settings, community centres, schools, or online platforms. Eligible articles included articles or reviews, all of which must be written in English. Grey literature was not included. Reference lists of included studies were also searched, and relevant articles extracted.

### 3.2. Search Strategy

Searches were carried out in June 2025. Databases included the following: MEDLINE, Scopus, Web of Science, CINAHL, and APA PsycNet. Interfaces were as follows:

- MEDLINE: OVID;
- Scopus: Elsevier;
- Web of Science: Clarivate;
- CINAHL Ultimate: EBSCOhost;
- APA PsycNet.

These databases were chosen for their extensive coverage of peer-reviewed work in this subject area. The query strings were developed with input from topic experts and included MeSH terms. Further details on the query strings are provided in the protocol (https://doi.org/10.17605/OSF.IO/WGV9T).

Two previously known articles were selected and confirmed to appear using the MEDLINE search string to validate the effectiveness of the query strings in locating the desired articles. Both articles were present in the test search.

### 3.3. Study/Source of Evidence Selection

**Figure 1:**
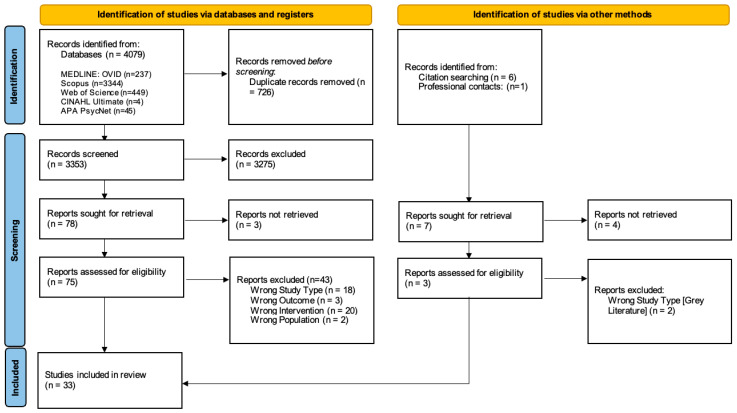
Preferred reporting items for systematic reviews and meta-analysis.

Search results were imported into Rayyan, a systematic review screening tool. Duplicates were removed, and remaining articles screened by two reviewers (EVS & MD) who independently included or excluded each article, first by title and abstract, followed by full text (see Figure 1). They were blinded to each other’s decisions to avoid bias. Decisions were tracked, and conflicts were resolved through discussion with another author outside of the screening team. Author, title, year, journal, and abstract are visible to reviewers during screening.

### 3.4. Data Extraction

Two human extractors worked sequentially. The first author (EVS) extracted the data, and another researcher (MD) reviewed the extraction for any errors or discrepancies. Any differences were resolved through discussion with the wider research team. The data extraction form is included (Supplementary Materials).

### 3.5. Quality Appraisal

Although critical appraisal is not typically required for scoping reviews, an MMAT [6] was conducted across the selected sources to ensure transparency of the data set and to adhere to the JBI guideline changes. Quality appraisal was initially carried out by one author (EVS) and peer reviewed by another (ABS).

The results indicate adequate methodological quality (see Table 1). The majority (n = 21) of the appraised studies were rated as Moderately High Quality. High Quality studies made up a smaller, but still significant portion (n = 6), and few (n = 3) were rated as Low or Very Low Quality. One non-primary paper was not appraised but was acknowledged as high quality and relevant.

## 4. Results

Thirty-three articles were analysed. The analysis follows typical expectations of a scoping review—a descriptive synthesis with narrative summary. Higher levels of interpretation are for the remit of systematic reviews, whereas this review aims to simply map the evidence.

Studies in this area predominantly use exclusively quantitative methods (69% of included studies), revealing potential experiential gaps. Because of this, samples tend to be larger, with 51% over 50 participants, which tend to be more generalisable. Studies took place in several countries, with the UK and Europe representing the main locations. Table 2 provides a detailed breakdown.

**Figure 2:**
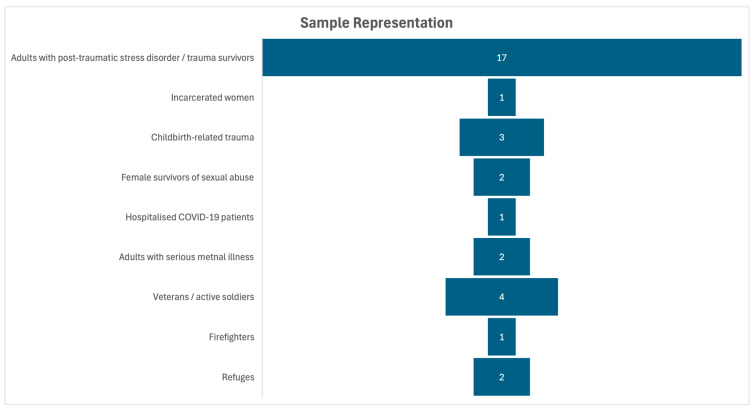
Sample representation.

The studies’ demographics are diverse, including adults with PTSD, incarcerated women, childbirth trauma survivors, female survivors of sexual abuse, hospitalised COVID-19 patients, adults with serious mental illness, veterans and active soldiers, firefighters, and refugees (See Figure 2). This breadth allows the results to be applicable to multiple real-world contexts.

**Figure 3:**
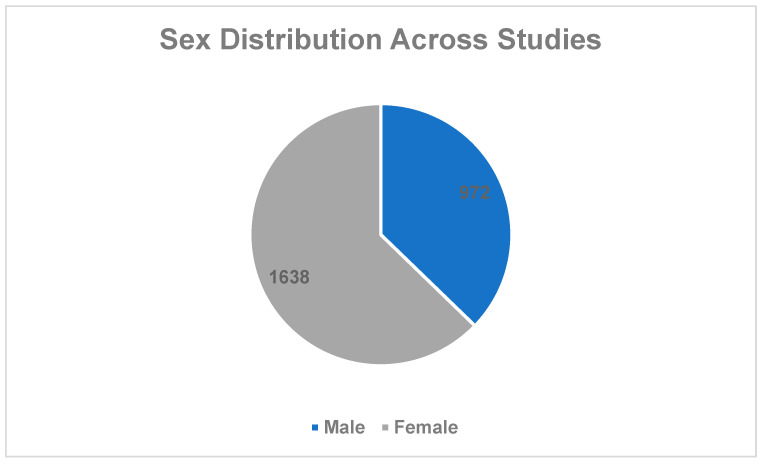
Sex distribution across included studies.

A total of 2610 participants had their sex reported, while two studies did not report sex for 120 participants. The majority of the studies primarily involved female participants. Seven investigations focused exclusively on women [10,12,13,27,30,33,39], whereas only one study included solely male participants [20]. The complete distribution of participant sex across all included studies is illustrated in Figure 3.

The most frequently studied interventions were EMDR, appearing 21 times (sometimes alone, often in combination with CBT, Prolonged Exposure (PE), or other therapies). CBT was also common, appearing 12 times in the data set (including subtypes like trauma-focused CBT, Cognitive Therapy for PTSD [CT-PTSD], and Cognitive Processing Therapy [CPT]). Prolonged Exposure (PE, n = 4) and Narrative Exposure Therapy (NET, n = 3) appeared less often but are still notable. Psychodynamic (n = 2), Hypnotherapy (n = 1), Trauma-informed yoga (n = 1), Schema therapy (n = 1), and various supportive approaches (counselling, stress management, etc.) each appear only once or twice, suggesting evidence gaps in this area. While EMDR and CBT were the most frequently studied interventions, this does not inherently indicate greater effectiveness. Conclusions about efficacy are based on the reported outcomes and study quality rather than the volume of research.

Both EMDR and (trauma-focused) CBT were found to be effective in reducing symptoms of trauma across various populations and contexts [7,8,9,12], but due to variability in study quality and contexts, larger controlled studies are needed [17]. Recovery rates had no significant difference, so choosing EMDR or CBT may depend on symptoms [7]. For example, EMDR was effective for military and emergency personnel [20,21], while CBT was widely used for general mental illness, trauma, and singular events like child loss [29,34,37].

While majorly successful, EMDR may not be effective for cases of chronic abuse-related PTSD (e.g., from intimate partner violence) [10]. That said, it may be more effective when combined with other therapeutic approaches such as PE [8], Cognitive Processing Therapy (CPT), or Eclectic Therapy, which foster a stronger therapeutic alliance, valuing supportive relational processes over deeper trauma work—which may be too challenging for this demographic [13]. Such therapies evidenced greater participation in social activities, improved emotional intimacy in relationships, better parenting relationships, increased engagement in hobbies and community life, greater occupational confidence, and higher life satisfaction [18]. This indicates a possible shift toward enhancing overall quality of life rather than focusing solely on symptom reduction.

Remote intensive programmes were less common but also had positive outcomes, with effects sustained at 6 months [8,14]. Thus, the evidence supports using tailored therapies, with treatment decisions influenced by symptoms, client preference, and culture to improve access to services. Brief psychotherapy methods (desensitisation, hypnotherapy, psychodynamic, schema) also met success in treating traumatic symptoms, particularly for complex and chronic trauma in specific cultural contexts [11,38,39] but a paucity in research prevented significant conclusions being drawn.

## 5. Discussion

Findings show EMDR and trauma-focused CBT are consistently effective, with increasing evidence for intensive/remote models and alternative therapies (schema therapy, NET, psychodynamic).

Across multiple contexts, EMDR and CBT emerged as the most extensively studied and effective interventions. The wider literature consolidates this, with EMDR being hailed as ‘the breakthrough therapy for overcoming anxiety, stress, and trauma’ [40,41]. Findings conclude EMDR to be equal, and in some cases superior, to CBT [19]. But a recent meta-analysis suggested trauma-focused CBT was slightly more effective than EMDR [42]. Achieving consensus on this subject remains challenging, indicating continued debate. However, EMDR may offer an advantage in terms of time efficiency, as positive outcomes are observed over fewer sessions than CBT [43]. When examining the relative efficacy across different populations, EMDR and CBT appear to demonstrate consistent effectiveness across a broad range of trauma presentations and demographic groups, though the evidence base is stronger for certain populations (e.g., adults with single-incident trauma) than others (e.g., older adults, incarcerated individuals).

Less traditional methods such as Narrative Exposure Therapy (NET), psychodynamic approaches, and schema therapy, also demonstrated promising results, particularly for specific populations or cultural contexts [11,38,39]. Comparing these alternative approaches to EMDR and CBT directly is challenging due to differences in study populations, outcome measures, and methodological quality. However, the available evidence suggests that while traditional approaches may show larger or more consistent effect sizes in controlled settings, alternative therapies may offer distinct advantages for specific populations—particularly those with complex trauma, cultural considerations, or where engagement with exposure-based methods is problematic. While some scholars believe them to be powerful for complex cases, especially where more traditional approaches have been unsuccessful [43], concerns remain [44]. Ethical concerns arise around the patient’s or therapist’s personality, the patient-therapist interaction, faulty therapy technique, or in the patient’s unresolvable social situation [45]. Controversially, some scholars also claim these therapies lack empirical support—notoriously stated by Medawar [46] and more recently echoed by Lieberman [47]. But this sentiment may be unfounded.

Opland and Torrico [48] noted that randomised controlled trials are viewed as the gold standard for empirical validation (which are typically used for CBT and EMDR), but their rigid structure often disadvantages alternative therapies, which are complex and individualised. Critics of these therapies frequently overlook how the validation process favours cognitive-behavioural approaches because they fit more easily into randomised trials. As a result, research on alternatives like psychodynamic therapy can seem less robust, making empirical validation challenging. Additionally, dynamic therapy may be particularly difficult to study because it is highly individualised and adapts to each patient’s needs, making it hard to standardise for research trials. This may explain why there is less research evidence for this approach. Additionally, broader patterns in research funding and evidence frameworks, which often prioritise short-term, manualised interventions, may potentially lead to less emphasis on relational or process-oriented therapies.

But Shedler [49] claimed that the distrust in psychodynamic therapy does not accord with available scientific evidence, thus may reflect biassed data selection. Shedler [49] draws on evidence such as Leichsenring and Leibing’s [50] meta-analysis that examined the efficacy of both psychodynamic psychotherapy and CBT for personality disorders, where the authors concluded that both treatments demonstrated effectiveness. Several articles corroborate this [51,52,53] as well as indicating that the psychological processes activated in psychodynamic therapy can contribute to lasting positive change beyond the conclusion of treatment. Emerging toolkit-based approaches like The Walters Method represent potential avenues for supporting long-term maintenance of therapeutic outcomes, particularly in complex trauma cases where this review identified evidence gaps. That said, TWM remains a less evaluated approach, warranting future empirical investigation.

Given the clear and notable benefits of EMDR and CBT and substantial gaps in knowledge, it may be worthwhile to further examine other psychotherapies for trauma-related distress, particularly in complex situations where intensive trauma-focused approaches could present significant challenges, e.g., for complex, chronic, or refugee-related trauma [13]. These findings are novel, emphasising that the type of intervention should not only be guided by efficacy but also by goals, comfort, and cultural and contextual compatibility between the person and intervention. TWM could be a good fit because it provides an accessible, less intimidating option for clients that does not require them to directly discuss their issues and instead uses ‘felt-sense’ psychotherapeutic techniques [54]. These techniques help transform traumatic memories and negative self-beliefs, leading to emotional freedom and mindfulness, and preparing clients for life after therapy.

### Major Research Gaps and Limitations

Although the results are promising, most studies were limited by small sample sizes or lack of control, so firm conclusions cannot be drawn. Several major research gaps constrain the generalisability of current findings. First, there is a notable absence of longitudinal evidence; most studies assess outcomes immediately post-treatment or at short-term follow-up (typically 3–6 months), with limited data on the durability of treatment effects beyond one year. This limitation is particularly significant given that trauma-related distress often follows a fluctuating course, and understanding long-term maintenance of gains is critical for evaluating true intervention effectiveness.

Second, small sample sizes characterise much of the research on alternative therapies and specific populations. This limits statistical power, increases the risk of Type II errors, and makes it difficult to detect meaningful differences between interventions or to identify moderators of treatment response. Studies examining schema therapy, psychodynamic approaches, and NET, while promising, are often based on samples of fewer than 50 participants, restricting confidence in effect estimates.

Third, cross-cultural validation remains severely limited. Most studies have been conducted in Western, high-income countries, with insufficient representation of diverse cultural contexts. Where cultural adaptations have been implemented (e.g., NET for refugee populations), results are encouraging, but systematic examination of how cultural factors moderate treatment effectiveness is lacking. This gap is particularly concerning given that trauma experiences, expressions of distress, help-seeking behaviours, and therapeutic relationships are all culturally mediated.

Finally, underrepresented populations, including incarcerated individuals, older adults, individuals with complex developmental trauma, and those with comorbid severe mental illness, remain inadequately studied. The evidence base for these groups is sparse, limiting our understanding of how trauma interventions should be adapted or selected for these vulnerable populations. Further, it is possible that grey literature or non-English studies may have more data on this.

Thus, this review focuses more on mapping the evidence and gap-spotting. Therefore, a reasonable direction for future research is to strengthen the evidence base in underrepresented populations, including incarcerated individuals, older adults, and those with complex trauma. Further validation would also be beneficial in reinforcing the effectiveness of emerging therapies such as schema therapy, remote programmes, and psychodynamic approaches. Additionally, future research should prioritise longitudinal designs with extended follow-up periods, adequately powered trials of alternative therapies, and systematic cross-cultural validation studies to enhance the generalisability and applicability of findings across diverse populations and settings.

## 6. Conclusions

This review contributes to the field by mapping the landscape of psychological therapies for PTSD, with particular attention to CBT and EMDR, as well as alternative approaches. EMDR and CBT are clearly demonstrated as the most studied and effective interventions. Alternative therapies (e.g., psychodynamic, schema, etc.) were identified in the literature, though a paucity of research prevented significant conclusions regarding their effectiveness, indicating further research would be valuable. Some evidence suggested that combination approaches (e.g., EMDR with CPT or eclectic therapy) showed benefits for quality-of-life outcomes in complex trauma cases. Intensive and remote formats also appeared in the literature as potential options for improving accessibility and time efficiency, though more research is needed. The evidence suggests that treatment selection should be guided by symptom profile, patient preference, and cultural considerations, recognising that no single intervention is universally superior. In other words, treatment should be tailored to each client’s needs rather than defaulting to the most studied approaches. Future research should address identified gaps in the evidence base, particularly regarding alternative therapies, complex trauma presentations, and diverse delivery formats.

## Figures and Tables

**Table 1:** Quality Appraisal.

Ref ID	First Author	Year	Appraisal
[7]	Belli	2025	Moderately High Quality
[8]	Bongaerts	2022	Moderately High Quality
[9]	Bryant	2013	Moderately High Quality
[10]	Colosetti	2000	Moderately High Quality
[11]	Brom	1989	Low Quality
[12]	Doherty	2025	Moderately High Quality
[13]	Edmond	2004	Moderately High Quality
[14]	Ellenbroek	2024	Moderately High Quality
[15]	Fan	2021	Moderately High Quality
[16]	Feingold	2018	Moderately High Quality
[17]	Forbes	1994	Moderately High Quality
[18]	Kehle-Forbes	2025	High Quality
[19]	Khan	2025	Moderate Quality
[20]	Kitchiner	2004	High Quality
[21]	Köhler	2017	Moderately High Quality
[22]	Krüger-Gottschalk	2025	Moderately High Quality
[23]	Lazrove	1998	Moderately High Quality
[24]	Melegkovits	2022	Moderately High Quality
[25]	Zepeda Méndez	2018	Moderately High Quality
[26]	Moghadam	2020	Moderate Quality
[27]	Mørkved	2018	High Quality
[28]	Motoziuk	2024	Moderate Quality
[29]	Mueser	2008	Moderately High Quality
[30]	Sandström	2006	Very Low Quality
[31]	Schubert	2016	Moderately High Quality
[32]	Susanty	2022	Moderately High Quality
[33]	Tarquinio	2012	Very Low Quality
[34]	Turrini	2021	The paper is not a primary study, thus cannot be appraised using the MMAT.
[35]	Wakusawa	2023	Moderate Quality
[36]	Van Woudenberg	2018	Moderately High Quality
[37]	Yu	2022	High Quality
[38]	Zinfandel	2024	High Quality
[39]	Lian	2024	Moderately High Quality

**Table 2:** Descriptive statistics of included articles.

Article Characteristics	N/33	Article Characteristics	N/32
**Type of evidence source**		**Sample size**	
Primary research article	32	<10	7
Secondary research article	1	10–30	6
**Methodology used**		31–50	3
Qualitative	5	51–70	4
Quantitative	23	71–100	6
Mixed Methods	4	>100	7
**Methods of data collection**		**Region**	
Multiple methods	4	UK and Ireland	4
Interviews	5	Europe	12
Surveys/questionaries	0	USA	6
Other	24	Asia	8
Not applicable	1	Australia	2
		Multiple	1

## Data Availability

The data presented in this study are available on request from the corresponding author due to additional screening data being stored on Rayyan, which requires a user login and cannot be openly shared.

## Supplementary Materials

The following supporting information can be downloaded at https://www.mdpi.com/article/10.3390/healthcare13233180/s1. Table S1: Data Extraction.

## Abbreviations

The following abbreviations are used in this manuscript:

TWM	The Walters Method
CBT	Cognitive Behavioural Therapy
EMDR	Eye Movement Desensitisation and Reprocessing
NET	Narrative Exposure Therapy
CPT	Cognitive Processing Therapy
CT-PTSD	Cognitive Therapy for PTSD
PE	Prolonged Exposure
